# Methyl 4-amino-2-chloro­pyrimidine-5-carboxyl­ate

**DOI:** 10.1107/S1600536814016080

**Published:** 2014-07-17

**Authors:** Ya-Ming Wu

**Affiliations:** a Department of Applied Chemistry, Nanjing College of Chemical Technology, Nanjing 210048, People’s Republic of China

**Keywords:** crystal structure

## Abstract

In the title compound, C_6_H_6_ClN_3_O_2_, all non-H atoms are approximately coplanar [maximum deviation = 0.012 (4) Å]; an intra­molecular N—H⋯O hydrogen bond occurs between the amino group and the carbonyl group. In the crystal, mol­ecules are linked by N—H⋯N hydrogen bonds into supra­molecular chains propagated along [101].

## Related literature   

For related structures, see: He & Kang (2006 ▶); He *et al.* (2007 ▶). For the synthesis, see: Ballard & Johnson (1942 ▶).
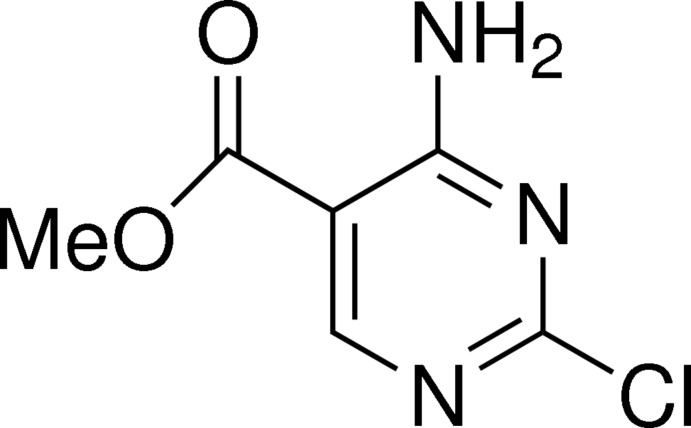


## Experimental   

### 

#### Crystal data  

C_6_H_6_ClN_3_O_2_
*M* *_r_* = 187.59
Monoclinic, 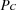

*a* = 3.9110 (8) Å
*b* = 10.136 (2) Å
*c* = 9.848 (2) Å
β = 98.71 (3)°
*V* = 385.89 (13) Å^3^
*Z* = 2
Mo *K*α radiation
μ = 0.45 mm^−1^
*T* = 293 K
0.20 × 0.20 × 0.15 mm

#### Data collection  

Enraf–Nonius CAD-4 diffractometer
1474 measured reflections
817 independent reflections
673 reflections with *I* > 2σ(*I*)
*R*_int_ = 0.041
3 standard reflections every 200 reflections intensity decay: 1%

#### Refinement  

*R*[*F* ^2^ > 2σ(*F* ^2^)] = 0.046
*wR*(*F* ^2^) = 0.109
*S* = 1.01
817 reflections
109 parameters
2 restraints
H-atom parameters constrained
Δρ_max_ = 0.25 e Å^−3^
Δρ_min_ = −0.36 e Å^−3^
Absolute structure: Flack (1983 ▶), 106 Friedel pairs
Absolute structure parameter: 0.07 (17)

### 

Data collection: *CAD-4 EXPRESS* (Enraf–Nonius, 1994 ▶); cell refinement: *CAD-4 EXPRESS*; data reduction: *XCAD4* (Harms & Wocadlo, 1995 ▶); program(s) used to solve structure: *SHELXTL* (Sheldrick, 2008 ▶); program(s) used to refine structure: *SHELXTL*; molecular graphics: *SHELXTL*; software used to prepare material for publication: *SHELXTL*.

## Supplementary Material

Crystal structure: contains datablock(s) I, New_Global_Publ_Block. DOI: 10.1107/S1600536814016080/xu5799sup1.cif

Structure factors: contains datablock(s) I. DOI: 10.1107/S1600536814016080/xu5799Isup2.hkl

Supporting information file. DOI: 10.1107/S1600536814016080/xu5799Isup3.cml

CCDC reference: 1013300

Additional supporting information:  crystallographic information; 3D view; checkCIF report

## Figures and Tables

**Table 1:** Hydrogen-bond geometry (Å, °)

*D*—H⋯*A*	*D*—H	H⋯*A*	*D*⋯*A*	*D*—H⋯*A*
N3—H3*A*⋯N2^i^	0.86	2.10	2.955 (7)	171
N3—H3*B*⋯O2	0.86	2.11	2.745 (7)	130

Symmetry code: (i) 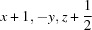.

**Fig. 1.**
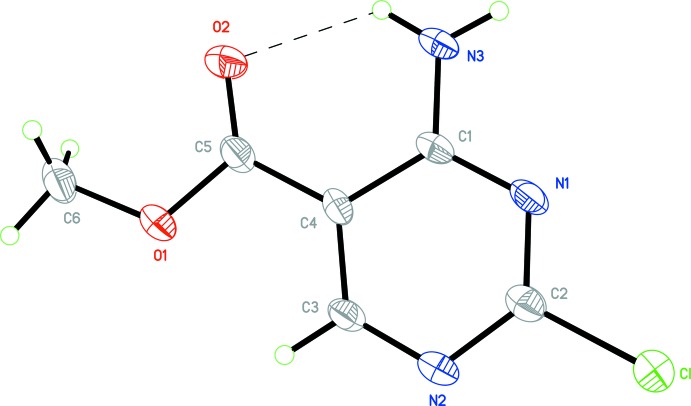
The molecular structure of (I), with the atom-numbering scheme. Displacement ellipsoids are drawn at the 50% probability level.

**Fig. 2.**
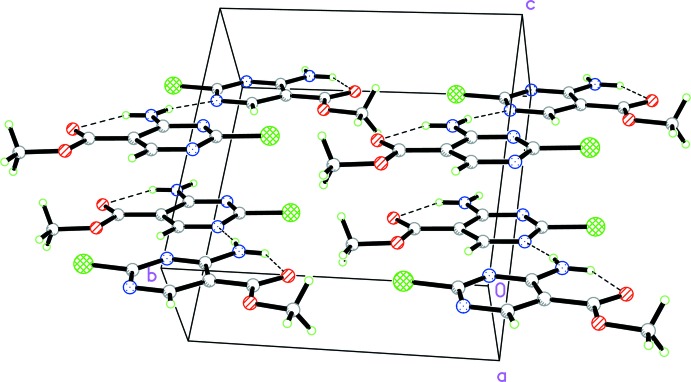
A packing diagram of (I).

## supplementary crystallographic information

### S1. Comment

The title compound,(I), is an intermediate for preparation of tuberculosis. We herein report its crystal structure.

The molecular structure of (I) is shown in Fig. 1. The bond lengths and angles are within normal ranges (He & Kang, 2006; He *et al.*, 2007). The pyrimidine ring is almost planar.

In the crystal, molecules are linked each other to form chains framework *via* intermolecular N—H···N hydrogen bonds, which with intramolecular N—H···O hydrogen bonds may be effective in the stabilization of the crystal structure.

### S2. Experimental

The title compound was synthesized according to the reported procedure (Ballard & Johnson, 1942). Crystals suitable for X-ray analysis were obtained by dissolving it (0.5 g) in dichloromethane (50 ml) and evaporating the solvent slowly at room temperature for about 5 d.

### S3. Refinement

H atoms were positioned geometrically with N—H = 0.86 and C—H = 0.93–0.96 Å, and refined in riding mode, U_iso_(H) = 1.5U_eq_(C) for methyl H atoms and 1.2U_eq_(C,N) for the others.

### Crystal data

**Table:**

C_6_H_6_ClN_3_O_2_	*F*(000) = 192
*M**_r_* = 187.59	*D*_x_ = 1.614 Mg m^−^^3^
Monoclinic, *P**c*	Melting point: 433 K
Hall symbol: p -2yc	Mo *K*α radiation, λ = 0.71073 Å
*a* = 3.9110 (8) Å	Cell parameters from 25 reflections
*b* = 10.136 (2) Å	θ = 9–13°
*c* = 9.848 (2) Å	µ = 0.45 mm^−^^1^
β = 98.71 (3)°	*T* = 293 K
*V* = 385.89 (13) Å^3^	Block, colorless
*Z* = 2	0.20 × 0.20 × 0.15 mm

### Data collection

**Table:**

Enraf–Nonius CAD-4 diffractometer	*R*_int_ = 0.041
Radiation source: fine-focus sealed tube	θ_max_ = 25.4°, θ_min_ = 2.0°
Graphite monochromator	*h* = 0→4
ω/2θ scans	*k* = −12→12
1474 measured reflections	*l* = −11→11
817 independent reflections	3 standard reflections every 200 reflections
673 reflections with *I* > 2σ(*I*)	intensity decay: 1%

### Refinement

**Table:**

Refinement on *F*^2^	Secondary atom site location: difference Fourier map
Least-squares matrix: full	Hydrogen site location: inferred from neighbouring sites
*R*[*F*^2^ > 2σ(*F*^2^)] = 0.046	H-atom parameters constrained
*wR*(*F*^2^) = 0.109	*w* = 1/[σ^2^(*F*_o_^2^) + (0.065*P*)^2^] where *P* = (*F*_o_^2^ + 2*F*_c_^2^)/3
*S* = 1.01	(Δ/σ)_max_ < 0.001
817 reflections	Δρ_max_ = 0.25 e Å^−^^3^
109 parameters	Δρ_min_ = −0.36 e Å^−^^3^
2 restraints	Absolute structure: Flack (1983), 106 Friedel pairs
Primary atom site location: structure-invariant direct methods	Absolute structure parameter: 0.07 (17)

### Special details

**Table:**

Geometry. All e.s.d.'s (except the e.s.d. in the dihedral angle between two l.s. planes) are estimated using the full covariance matrix. The cell e.s.d.'s are taken into account individually in the estimation of e.s.d.'s in distances, angles and torsion angles; correlations between e.s.d.'s in cell parameters are only used when they are defined by crystal symmetry. An approximate (isotropic) treatment of cell e.s.d.'s is used for estimating e.s.d.'s involving l.s. planes.
Refinement. Refinement of *F*^2^ against ALL reflections. The weighted *R*-factor *wR* and goodness of fit *S* are based on *F*^2^, conventional *R*-factors *R* are based on *F*, with *F* set to zero for negative *F*^2^. The threshold expression of *F*^2^ > σ(*F*^2^) is used only for calculating *R*-factors(gt) *etc*. and is not relevant to the choice of reflections for refinement. *R*-factors based on *F*^2^ are statistically about twice as large as those based on *F*, and *R*- factors based on ALL data will be even larger.

### Fractional atomic coordinates and isotropic or equivalent isotropic displacement parameters (Å^2^)

**Table:**

	*x*	*y*	*z*	*U*_iso_*/*U*_eq_	
Cl	1.0210 (5)	−0.25325 (18)	0.5060 (2)	0.0602 (4)	
O1	0.6389 (12)	0.3421 (3)	0.3059 (4)	0.0559 (11)	
C1	1.1205 (14)	0.1186 (6)	0.5586 (5)	0.0363 (14)	
N1	1.1550 (12)	−0.0104 (6)	0.5759 (4)	0.0411 (11)	
O2	0.9976 (15)	0.3988 (3)	0.4968 (5)	0.0597 (12)	
C2	0.9749 (16)	−0.0851 (5)	0.4819 (5)	0.0399 (13)	
N2	0.7645 (13)	−0.0485 (4)	0.3675 (5)	0.0423 (12)	
N3	1.3017 (15)	0.1949 (5)	0.6544 (5)	0.0480 (12)	
H3A	1.4322	0.1594	0.7229	0.058*	
H3B	1.2878	0.2793	0.6476	0.058*	
C3	0.7362 (14)	0.0804 (4)	0.3525 (5)	0.0381 (13)	
H3C	0.5923	0.1118	0.2754	0.046*	
C4	0.9020 (14)	0.1715 (5)	0.4414 (5)	0.0339 (12)	
C5	0.8591 (15)	0.3139 (6)	0.4214 (6)	0.0409 (13)	
C6	0.576 (2)	0.4789 (6)	0.2751 (8)	0.0626 (18)	
H6A	0.4174	0.4871	0.1911	0.094*	
H6B	0.7904	0.5215	0.2650	0.094*	
H6C	0.4795	0.5199	0.3485	0.094*	

### Atomic displacement parameters (Å^2^)

**Table:**

	*U*^11^	*U*^22^	*U*^33^	*U*^12^	*U*^13^	*U*^23^
Cl	0.0703 (9)	0.0469 (7)	0.0553 (8)	−0.0003 (8)	−0.0160 (6)	0.0032 (7)
O1	0.067 (3)	0.051 (2)	0.040 (2)	0.008 (2)	−0.023 (2)	0.000 (2)
C1	0.031 (3)	0.046 (3)	0.029 (3)	−0.002 (3)	−0.006 (2)	−0.003 (2)
N1	0.041 (3)	0.052 (3)	0.026 (2)	0.002 (2)	−0.008 (2)	0.000 (2)
O2	0.070 (3)	0.046 (2)	0.053 (2)	−0.008 (3)	−0.023 (2)	−0.007 (2)
C2	0.039 (3)	0.050 (3)	0.030 (3)	0.003 (3)	0.002 (3)	−0.003 (2)
N2	0.041 (3)	0.051 (3)	0.031 (2)	−0.003 (2)	−0.008 (2)	−0.003 (2)
N3	0.057 (3)	0.049 (2)	0.031 (2)	−0.004 (3)	−0.016 (2)	−0.004 (2)
C3	0.039 (3)	0.046 (3)	0.026 (3)	0.001 (3)	−0.006 (2)	−0.003 (3)
C4	0.032 (3)	0.040 (3)	0.028 (3)	0.006 (2)	−0.003 (2)	0.006 (2)
C5	0.040 (3)	0.048 (3)	0.032 (3)	0.004 (3)	−0.005 (2)	0.004 (2)
C6	0.060 (4)	0.051 (3)	0.067 (4)	0.008 (3)	−0.023 (3)	0.012 (3)

### Geometric parameters (Å, º)

**Table:**

Cl—C2	1.726 (5)	N2—C3	1.318 (6)
O1—C5	1.350 (7)	N3—H3A	0.8600
O1—C6	1.433 (7)	N3—H3B	0.8600
C1—N1	1.324 (8)	C3—C4	1.367 (7)
C1—N3	1.337 (7)	C3—H3C	0.9300
C1—C4	1.432 (7)	C4—C5	1.464 (7)
N1—C2	1.315 (7)	C6—H6A	0.9600
O2—C5	1.210 (7)	C6—H6B	0.9600
C2—N2	1.343 (8)	C6—H6C	0.9600
			
C5—O1—C6	116.7 (5)	C4—C3—H3C	117.5
N1—C1—N3	116.6 (5)	C3—C4—C1	115.5 (4)
N1—C1—C4	120.7 (5)	C3—C4—C5	123.1 (4)
N3—C1—C4	122.7 (5)	C1—C4—C5	121.3 (4)
C2—N1—C1	116.4 (5)	O2—C5—O1	122.4 (5)
N1—C2—N2	128.8 (5)	O2—C5—C4	126.0 (5)
N1—C2—Cl	116.0 (4)	O1—C5—C4	111.6 (4)
N2—C2—Cl	115.2 (4)	O1—C6—H6A	109.5
C3—N2—C2	113.5 (5)	O1—C6—H6B	109.5
C1—N3—H3A	120.0	H6A—C6—H6B	109.5
C1—N3—H3B	120.0	O1—C6—H6C	109.5
H3A—N3—H3B	120.0	H6A—C6—H6C	109.5
N2—C3—C4	125.0 (5)	H6B—C6—H6C	109.5
N2—C3—H3C	117.5		
			
N3—C1—N1—C2	179.6 (6)	N3—C1—C4—C3	179.6 (6)
C4—C1—N1—C2	−1.1 (9)	N1—C1—C4—C5	179.5 (5)
C1—N1—C2—N2	1.9 (10)	N3—C1—C4—C5	−1.3 (8)
C1—N1—C2—Cl	−179.8 (4)	C6—O1—C5—O2	0.4 (10)
N1—C2—N2—C3	−1.5 (10)	C6—O1—C5—C4	−179.9 (6)
Cl—C2—N2—C3	−179.9 (5)	C3—C4—C5—O2	179.7 (7)
C2—N2—C3—C4	0.5 (8)	C1—C4—C5—O2	0.6 (9)
N2—C3—C4—C1	0.0 (8)	C3—C4—C5—O1	0.0 (8)
N2—C3—C4—C5	−179.2 (6)	C1—C4—C5—O1	−179.1 (6)
N1—C1—C4—C3	0.4 (8)		

### Hydrogen-bond geometry (Å, º)

**Table:**

*D*—H···*A*	*D*—H	H···*A*	*D*···*A*	*D*—H···*A*
N3—H3*A*···N2^i^	0.86	2.10	2.955 (7)	171
N3—H3*B*···O2	0.86	2.11	2.745 (7)	130

Symmetry code: (i) *x*+1, −*y*, *z*+1/2.
